# Supplementary material for: OpenMM 7: Rapid development of high performance algorithms for molecular dynamics
Source: PLoS Comput Biol. 2017 Jul 26;13(7):e1005659. doi: 10.1371/journal.pcbi.1005659 (PMC5549999; doi:10.1371/journal.pcbi.1005659)
Supplement: S1 Source Code — (ZIP) [file pcbi.1005659.s002.zip › OpenMM-7.0.1-Source/docs/api-python/generated/simtk.openmm.openmm.AmoebaWcaDispersionForce.html]

AmoebaWcaDispersionForce — OpenMM 7.0.1.dev-5e86c4f documentation


# AmoebaWcaDispersionForce¶

*class* `simtk.openmm.openmm.``AmoebaWcaDispersionForce`(*\*args*)¶
:   This class implements a nonbonded interaction between pairs of particles typically used along with AmoebaGeneralizedKirkwoodForce as part of an implicit solvent model.

    To use it, create an AmoebaWcaDispersionForce object then call addParticle() once for each particle. After a particle has been added, you can modify its force field parameters by calling setParticleParameters(). This will have no effect on Contexts that already exist unless you call updateParametersInContext().

    `__init__`(*self*) → AmoebaWcaDispersionForce¶
    :   \_\_init\_\_(self, other) -> AmoebaWcaDispersionForce

        Create an AmoebaWcaDispersionForce.

    Methods

    |  |  |
    | --- | --- |
    | `__init__`((self) -> AmoebaWcaDispersionForce) | \_\_init\_\_(self, other) -> AmoebaWcaDispersionForce |
    | `addParticle`((self, radius, epsilon) -> int) | Set the force field parameters for a WCA dispersion particle. |
    | `getAwater`((self) -> double) |  |
    | `getDispoff`((self) -> double) |  |
    | `getEpsh`((self) -> double) |  |
    | `getEpso`((self) -> double) |  |
    | `getForceGroup`((self) -> int) | Get the force group this Force belongs to. |
    | `getNumParticles`((self) -> int) | Get the number of particles |
    | `getParticleParameters`(self, particleIndex) | Get the force field parameters for a WCA dispersion particle. |
    | `getRminh`((self) -> double) |  |
    | `getRmino`((self) -> double) |  |
    | `getShctd`((self) -> double) |  |
    | `getSlevy`((self) -> double) |  |
    | `setAwater`(self, inputValue) |  |
    | `setDispoff`(self, inputValue) |  |
    | `setEpsh`(self, inputValue) |  |
    | `setEpso`(self, inputValue) |  |
    | `setForceGroup`(self, group) | Set the force group this Force belongs to. |
    | `setParticleParameters`(self, particleIndex, ...) | Set the force field parameters for a WCA dispersion particle. |
    | `setRminh`(self, inputValue) |  |
    | `setRmino`(self, inputValue) |  |
    | `setShctd`(self, inputValue) |  |
    | `setSlevy`(self, inputValue) |  |
    | `updateParametersInContext`(self, context) | Update the per-particle parameters in a Context to match those stored in this Force object. |
    | `usesPeriodicBoundaryConditions`((self) -> bool) | Returns whether or not this force makes use of periodic boundary conditions. |

    `getNumParticles`(*self*) → int¶
    :   Get the number of particles

    `setParticleParameters`(*self*, *particleIndex*, *radius*, *epsilon*)¶
    :   Set the force field parameters for a WCA dispersion particle.

        |  |  |
        | --- | --- |
        | Parameters: | - **particleIndex** (*int*) – the particle index - **radius** (*double*) – radius - **epsilon** (*double*) – epsilon |

    `getParticleParameters`(*self*, *particleIndex*)¶
    :   Get the force field parameters for a WCA dispersion particle.

        |  |  |
        | --- | --- |
        | Parameters: | **particleIndex** (*int*) – the particle index |
        | Returns: | - **radius** (*double*) –   radius - **epsilon** (*double*) –   epsilon |

    `addParticle`(*self*, *radius*, *epsilon*) → int¶
    :   Set the force field parameters for a WCA dispersion particle.

        |  |  |
        | --- | --- |
        | Parameters: | - **radius** (*double*) – radius - **epsilon** (*double*) – epsilon |
        | Returns: | index of added particle |
        | Return type: | int |

    `updateParametersInContext`(*self*, *context*)¶
    :   Update the per-particle parameters in a Context to match those stored in this Force object. This method provides an efficient method to update certain parameters in an existing Context without needing to reinitialize it. Simply call setParticleParameters() to modify this object’s parameters, then call updateParametersInContext() to copy them over to the Context.

        The only information this method updates is the values of per-particle parameters. All other aspects of the Force are unaffected and can only be changed by reinitializing the Context.

    `getEpso`(*self*) → double¶

    `getEpsh`(*self*) → double¶

    `getRmino`(*self*) → double¶

    `getRminh`(*self*) → double¶

    `getAwater`(*self*) → double¶

    `getShctd`(*self*) → double¶

    `getDispoff`(*self*) → double¶

    `getSlevy`(*self*) → double¶

    `setEpso`(*self*, *inputValue*)¶

    `setEpsh`(*self*, *inputValue*)¶

    `setRmino`(*self*, *inputValue*)¶

    `setRminh`(*self*, *inputValue*)¶

    `setAwater`(*self*, *inputValue*)¶

    `setShctd`(*self*, *inputValue*)¶

    `setDispoff`(*self*, *inputValue*)¶

    `setSlevy`(*self*, *inputValue*)¶

    `usesPeriodicBoundaryConditions`(*self*) → bool¶
    :   Returns whether or not this force makes use of periodic boundary conditions.

        |  |  |
        | --- | --- |
        | Returns: | true if nonbondedMethod uses PBC and false otherwise |
        | Return type: | bool |

    `__delattr__`¶
    :   x.\_\_delattr\_\_(‘name’) <==> del x.name

    `__format__`()¶
    :   default object formatter

    `__getattribute__`¶
    :   x.\_\_getattribute\_\_(‘name’) <==> x.name

    `__hash__`¶

    `__reduce__`()¶
    :   helper for pickle

    `__reduce_ex__`()¶
    :   helper for pickle

    `__sizeof__`() → int¶
    :   size of object in memory, in bytes

    `__str__`¶

    `getForceGroup`(*self*) → int¶
    :   Get the force group this Force belongs to.

    `setForceGroup`(*self*, *group*)¶
    :   Set the force group this Force belongs to.

        |  |  |
        | --- | --- |
        | Parameters: | **group** (*int*) – the group index. Legal values are between 0 and 31 (inclusive). |

High performance molecular simulation on GPUs


### Navigation

- Application Layer
- Library Layer
  - Core Objects
  - Forces
  - Integrators
  - Extras

©2015, Stanford University and the Authors.
|
Powered by Sphinx 1.3.1
& Alabaster 0.7.7
|
Page source
